# Prognostic factors for ARDS: clinical, physiological and atypical immunodeficiency

**DOI:** 10.1186/s12890-020-1131-0

**Published:** 2020-04-23

**Authors:** Min Song, Yijie Liu, Zhiwen Lu, Hong Luo, Hong Peng, Ping Chen

**Affiliations:** 1 Department of Pulmonary and Critical Care Medicine, The Second Xiangya Hospital, Central South University, No.139 Renmin Road, Changsha, 410011 Hunan China; 2 The Respiratory Disease Research Institute of Hunan Province; The Respiratory Disease Diagnosis and Treatment Center of Hunan Province, No.139 Renmin Road, Changsha, 410011 Hunan China; 3 School of Mathematics and Statistics, Hunan Normal University, Changsha, 410081 Hunan China

**Keywords:** Acute respiratory distress syndrome, Morbidity, Outcome assessment, Prognostic

## Abstract

**Background:**

Risk factors affecting the prognosis of acute respiratory distress syndrome (ARDS) in adults were investigated. The aim was to identify new predictors for ARDS patient prognosis, including those with clinical, pathophysiological, and atypical immunodeficiency.

**Methods:**

ARDS patients were retrospectively included. The patients were grouped and analysed according to different oxygenation index grades and prognosis, and factors influencing prognosis and survival were examined. Adolescent patients, patients with typical immunodeficiency and patients who died within 24 h after being diagnosed with ARDS were excluded. The predictive value for mortality was determined by Cox proportional hazard analysis.

**Results:**

In total, 201 patients who fulfilled the Berlin definition of ARDS were included. The severity of critical illness on the day of enrolment, as measured by the Acute Physiology and Chronic Health Evaluation (APACHE) II score (*P* = 0.016), Sequential Organ Failure Assessment (SOFA) score (*P* = 0.027), and PaO_2_/FiO_2_ (*P* = 0.000), worsened from mild to severe ARDS cases. Compared with survivors, non-survivors were significantly older and had higher APACHE II and SOFA scores. Moreover, significantly lower lymphocyte/neutrophil ratios and leukocyte counts were found among non-survivors than survivors (*P* = 0.008, *P* = 0.012). A moderate positive correlation between the lymphocyte/neutrophil and PaO_2_/FiO_2_ ratios (*P* = 0.023) was observed. In predicting 100-day survival in patients with ARDS, the area under the curve (AUC) for the lymphocyte/neutrophil ratio was significantly higher than those for the PaO_2_/FiO_2_ ratio alone, body mass index (BMI) alone, and the lymphocyte count alone (*P* = 0.0062, 0.0001, and 0.0154). Age (per log_10_ years), BMI < 24, SOFA score, leukocyte count, and the lymphocyte/neutrophil ratio were independent predictors of 28-day mortality in ARDS patients. Additionally, ARDS patients with a lymphocyte/neutrophil ratio < 0.0537 had increased 28-day mortality rates (*P* = 0.0283). Old age affected both 28-day and 100-day mortality rates (*P* = 0.0064,0.0057).

**Conclusions:**

Age (per log_10_ years), BMI < 24, SOFA score, lymphocytes, and the lymphocyte/neutrophil ratio were independent predictors of 100-day mortality in patients with ARDS. The lymphocyte/neutrophil ratio may represent a potential molecular marker to evaluate atypical immunosuppression or impairment in patients with ARDS.

## Background

Acute respiratory distress syndrome (ARDS) is a life-threatening respiratory disease with a high mortality rate in critically ill patients [1, 2]. Although many in-depth studies on ARDS have been conducted, the specific pathogenesis and prognostic factors of the disease remain unclear. Indeed, despite improvements in ventilatory techniques and extensive research to date, ARDS continues to be associated with high mortality [3, 4].

Nonetheless, clinical and animal studies have shown that the activation of multiple inflammatory cells and the release of inflammatory mediators play important roles in the development and outcome of ARDS [5]. The involvement of immune cells, including neutrophils [6, 7] lymphocytes [8], and regulatory T-cell [9, 10], has become an active topic of research in ARDS pathogenesis. To date, few clinical studies on the immune status of ARDS patients have focused on aetiology, treatment and prognosis [11]. Immunocompromised individuals represent a significant proportion of ARDS patients [11, 12], these patients do not have the ability to respond normally to an infection due to an impaired or weakened immune system. Some studies have shown that ARDS occurs in patients with previous immunodeficiencies, such as haematologic malignancies, active solid tumours, solid organ transplantation, and acquired immunodeficiency syndrome, as well as in patients taking long-term or high-dose corticosteroids or immunosuppressants, and those who use extra-corporeal membrane oxygenation (ECMO) may have a better prognosis [13]. Compared with immunocompetent subjects, ARDS patients with typical immunodeficiency have higher mortality regardless of ARDS severity [11]. In addition, atypical immunosuppression is frequently found among ARDS patients, and virus infections are also increasingly being reported in ARDS patients without typical immunosuppression [13]. Overall, there is currently a lack of uniform molecular markers for patients with atypical immunosuppression or impaired status. Furthermore, it is not well known whether the status of atypical immunodeficiency affects the prognosis of ARDS. Managing patients with atypical immunosuppression in intensive care unit (ICU) can be challenging, updated epidemiological and outcomes studies are needed to evaluate the condition of these patients.

This aim of our study was to identify a convenient and easy-to-use molecular biomarker to detect and evaluate the status of patients with atypical immunosuppression in ARDS patients.

## Methods

### Study design and patients

This retrospective observational cohort study was conducted with ARDS patients hospitalized in the critical care centre of a university-based tertiary care hospital (The Second Xiangya Hospital of Central South University) in Hunan, China, from January 2011 to August 2018. Institutional approval was provided by the Second Xiangya Hospital of Central South University Biomedical Research Ethics Committee (Hunan, China). Written informed consent was waived because of the retrospective observational design. All patient data were anonymously recorded to ensure confidentiality.

### Inclusion and exclusion criteria

Patients admitted to the critical care centre with a diagnosis of ARDS based on the 2012 Berlin definition [14] were included in our study if they met the inclusion criteria and none of the exclusion criteria. All eligible patients were over 18 years old with available neutrophil and lymphocyte count results within 24 h after ICU admission. Patients who were repeatedly admitted to the ICU, lack of neutrophil and lymphocyte records, had chronic haematological disorders, were under the age of 18 years, or died within 24 h of receiving a diagnosis of ARDS were excluded. In addition, we excluded patients who were defined as having an immunodeficiency with the following aetiologies: (1) haematological malignancies, (2) active solid tumours or specific anti-tumour treatment within a year, (3) solid organ transplant, (4) acquired immunodeficiency syndrome (AIDS), or (5) long-term or high-dose corticosteroid (CS) or immunosuppressant (IS) therapy. Long-term CS therapy was defined as > 7.5 mg of prednisone/day for > 3 months, and a high dose was defined as > 1 mg/kg for > 1 week within the previous 3 months. According to the PaO_2_/FiO_2_ ratio, patients were categorized as mild (200 mmHg<PaO_2_/FiO_2_ ≤ 300 mmHg, *n* = 31), moderate (100 mmHg<PaO_2_/FiO_2_ ≤ 200 mmHg, *n* = 61), and severe (PaO_2_/FiO_2_ ≤ 100 mmHg, *n* = 109) groups. In addition, 201 patients were included and divided into a survivor group (*n* = 80) and a non-survivor group (*n* = 121) according to the final clinical results.

### Data extraction and outcome

Demographic and baseline characteristics such as age, sex, body mass index (BMI), ARDS risk factors, severity of illness upon admission to the ICU (Acute Physiology and Chronic Health Evaluation (APACHE) II score) [15], and the Sequential Organ Failure Assessment (SOFA) score [16] were recorded and analysed. We recorded routine blood examination results within 24 h and within 3 days after ICU admission. Two authors completed the data collection independently. The primary outcome was mortality, and the secondary outcomes were ICU mortality and hospital mortality. We also calculated 28-day mortality and 100-day mortality rates.

### Blood measurements and flow cytometric analysis

The white blood cell count, C-reactive protein (CRP) level and procalcitonin (PCT) level were measured. Serum levels of haemoglobin, albumin, immunoglobulins (IgG, IgA, IgM, IgE), and complement components (C3, C4) (R&D Systems, USA) was determined by enzyme-linked immunosorbent assay (ELISAs) in accordance with the manufacturer’s instructions. To analyse T-lymphocytes, cell staining kit (BD ingen™, USA) was used to detect CD4 + CD8 + CD3 + cells according to the manufacturer’s protocol. Briefly, peripheral blood mononuclear cells (PBMCs) were incubated with a mixture of luciferin isothiocyanate anti-CD4 and apc anti-CD8 at 4 °C for 30 min. Facscalibur flow cytometry (BD Biosciences, USA) and CellQuest software (BD Biosciences, USA) were used for flow cytometry analysis. A homotype control was used to ensure antibody specificity [17, 18].

### Statistical analysis

We used the Kolmogorov-Smirnov test to assess distribution normality. The continuous variables were reported as the mean ± SD or median (IQR). An independent samples t-test was used to evaluate normally distributed data, and the Mann-Whitney test was employed to evaluate non-normally distributed data when comparing two groups. In multi-group comparisons, one-way ANOVA and Kruskal-Wallis test were used to analyze the normal and non-normal distribution data respectively, and the *P*-values adjusted by Bonferroni were used for multiple groups of comparison. Classification data number (percentage) aggregation, and Chi-square or Fisher’s exact test. Spearman’s rank correlation was adopted to determine correlations among variables. Area under the curve operating characteristic (ROC) was used to evaluate the prognostic value of the subject properties, diagnostic and test parameters. The cut-off point was obtained by determining the optimal den index (sensitivity+specificity-1). We used Kaplan-Meier plots and log-rank tests to compare the survival rate of each group. To calculate an independent predictor of mortality of 100 days, with a stepwise binary logistic regression variables for the regression values ​​of *P* < 0.05 (one variable was entered when P < 0.05, and one was deleted when *P* > 0.10). The odds ratio (OR), *P*-value and 95% CI were used to represent results. All tests were double-tailed, and P < 0.05 was considered statistically significant. All analyses were performeded using IBM SPSS 22.0 and MedCalc v.11.0.

## Results

### Baseline characteristics and patient outcome

A total of 201 patients meeting the Berlin definition of ARDS from January 2011 to August 2018 were included in this study. Characteristics at enrolment and outcomes of the study population are shown in Table 1 and Table 2. There were no statistically significant differences in age, sex or BMI among the mild, moderate and severe ARDS groups. The most common aetiologies of ARDS were pneumonia, sepsis and pancreatitis. According to the APACHE II score (*P* = 0.016), SOFA score (*P* = 0.027), and PaO_2_/FiO_2_ (*P* = 0.000), it can be seen that the severity of critical illness on the day of enrolment worsened from mild to severe ARDS, as shown in Tables 1 and 2. The 100-day mortality rate for patients with ARDS was 60.2% (121/201). Compared to non-survivors, the survivors were significantly younger, with relatively low scores for APACHE II and SOFA. Survivors had higher BMIs and PaO_2_/FiO_2_ ratios than did non-survivors.1 2

**Table 1:** Baseline characteristics of the enrolled study population. Normally distributed quantitative data are expressed as means±standard deviation. Non-normally distributed quantitative data are expressed as medians (IQR)

Variables	Acute respiratory distress syndrome				^**∆**^P-value
	Total	Mild	Moderate	Severe	
Number	201	31	61	109	
Age,years	54.24 ± 16.35	55.38 ± 15.60	52.43±	54.93 ± 16.03	0.579
Sex,male/female,n	130/71	19:12	40/21	71/38	0.415
BMI,kg/m^2^	23.90 ± 3.82	24.22 ± 3.93	23.83 ± 4.05	23.90 ± 3.82	0.896
Cause of ARDS					
Pneumonia	125	19	37	69	
Non-pulmonary spesis	38	6	15	17	
Pancreatitis	15	4	7	4	
Trauma	7	0	0	7	
Aspiration	2	0	0	2	
Others	14	2	2	10	
APACHE II score	14.19(7.69 to 31.00)	12.74(7.69 to 24.37)	14.52 (9.86 to 27.69)	14.34 (9.68 to 31.00)*	0.016
SOFA score	4.98(4.65 to 5.30)	4.59(3.97 to 5.20)	5.11 (4.71 to 5.53)	5.26 (4.89 to 5.63)	0.027
PaO_2_/FiO_2,_mmHg	123 (112 to 134)	280 (267 to 294)	140 (132 to 146)*	69 (65 to 72)* ^#^	0.000
CRP,mg/L	129.82 (129.82 to 169.91)	110.09 (76.59 to 143.59)	163.90 (138.67 to 189.14)*	203.33 (115.97 to 290.68)*	0.011
PCT,ng/ml	12.20 (8.12 to 16.29)	9.37 (5.45 to 13.30)	10.15 (1.51 to 18.80)	17.05 (7.58 to 27.73)*	0.175
Albumin,g/L	27.67 ± 5.82	27.13 ± 3.17	27.07 ± 6.05	28.10 ± 6.05^*^	0.759
Hemoglobin,g/L	107.67 ± 28.32	104.04 ± 23.76	107.29 ± 28.05	108.82 ± 29.73	0.759
Leukocytes,10^9^/L	11.33(10.12 to 12.54)	10.98 (8.67 to 13.28)	10.91 (9.62 to 12.22)	13.65 (8.65 to 18.64)	0.298
Lymphocytes,10^9^/L	1.10 (0.95 to 1.26)	1.28 (0.86 to 1.75)	1.10 (8.67 to 13.28)	1.07 (0.87 to 1.27)*	0.025
Neutrophils,10^9^/L	10.78 (8.38 to 13.18)	8.35 (6.91 to 9.78)	11.66 (7.59 to 15.74)	13.03 (7.31 to 18.76)	0.371
Lymphocyte/Neutrophil ratio	0.19 ± 0.03	0.35 ± 0.23	0.17 ± 0.03^*^	0.15 ± 0.02^*^	0.001
Virus infection,n,%	23(11.44%)	1(3.23%)	10(16.39%)	12(11.0%)	
28-day mortality,n,%	103(51.24%)	13(41.94%)	31(50.82%)	59(54.13%)	
100-day mortality,n,%	121(60.20%)	15(48.39%)	37(60.66%)	69(63.30%)	
Immunoglobulin					
IgG,g/L	10.87 ± 1.86	11.71 ± 4.96	10.82 ± 1.94	10.59 ± 5.36	0.095
IgA,g/L	1.98 ± 0.24	1.92 ± 1.09	1.85 ± 0.72	2.11 ± 1.40	0.270
IgM,g/L	1.05 ± 0.86	1.32 ± 1.18	1.02 ± 0.92	1.00 ± 0.72	0.598
IgE,ng/mL	620.36 ± 145.11	1426.20 ± 119.89	640.55 ± 32.45	356.87 ± 78.73^*^	0.025
Complement components					
C3	5.62 ± 1.78	12.27 ± 1.83	7.13 ± 3.14	0.84 ± 0.33^*^	0.186
C4	1.33 ± 0.58	0.25 ± 0.13	0.24 ± 0.11	2.25 ± 0.95^*^	0.663
T-lymphocytes subsets					
CD3+ cells,%	62.75 ± 15.29	71.00 ± 13.78	63.35 ± 12.44	60.62 ± 17.11	0.229
CD4+ cells,%	35.15 ± 15.48	36.40 ± 13.69	34.10 ± 11.14	35.66 ± 8.47	0.951
CD4+ cells count (PCS/ul)	383.20 ± 67.18	176.50 ± 67.18	383.67 ± 94.79	434.08 ± 78.66	0.708
CD8+ cells,%	27.33 ± 13.46	34.33 ± 6.37	25.60 ± 11.59	26.92 ± 14.01	0.275
CD8+ cells count (PCS/ul)	212.33 ± 64.30	134.00 ± 30.63	286.67 ± 51.72	178.91 ± 70.96	0.409
CD4+/CD8+ ratio	1.95 ± 0.26	1.29 ± 0.23	1.91 ± 0.93	2.14 ± 0.48	0.623
B-lymphocytes					
B-lymphocytes,%	30.25 ± 17.28	18.00 ± 3.24	26.20 ± 7.81	33.36 ± 7.81	0.598
B-lymphocytes cell count (PCS/μl)	301.05 ± 73.77	69.01 ± 8.89	413.20 ± 41.42	271.18 ± 78.37	0.665
NK cell					
NK cells,%	8.76 ± 5.91	17.00 ± 4.41	9.40 ± 0.81	7.73 ± 4.58	0.331
NK cell count (PCS/ul)	68.77 ± 46.46	68.20 ± 7.66	82.60 ± 14.82	62.55 ± 15.15	0.751

Qualitative data are presented as numbers (%). ^∆^*P*-value for the three groups (mild, moderate, and severe ARDS groups); *P < 0.05 versus mild ARDS; ^#^*P* < 0.05 versus moderate ARDS. *BMI* body mass index, *APACHE* Acute Physiology and Chronic Health Evaluation *SOFA* Sequential Organ Failure Assessment, *CRP* C-reactive protein, *PCT* procalcitonin

**Table 2:** Comparison of clinical characteristics of ARDS patients according to survival status. Normally distributed quantitative data are expressed as means±standard deviation. Non-normally distributed quantitative data are expressed as medians (IQR)

Variables	Non-survivors(***n*** = 121)	Survivors(***n*** = 80)	***P***-value
Age,years	56.96 ± 17.09	50.16 ± 14.33	0.004
Sex,male/female,n	76/45	54/26	0.496
BMI,kg/m^2^	23.41 ± 3.88	24.69 ± 3.93	0.027
APACHE II score	14.96(14 to 16)	13.51 (13 to 15)	0.036
SOFA score	5.42 (5 to 6)	4.15 (4 to 5)	0.000
PaO_2_/FiO_2_,mmHg	115 (102 to 188)	135 (115 to 154)	0.042
CRP,mg/L	152.20 (126.50 to 177.89)	146.32 (113.27 to 179.37)	0.778
PCT,ng/Ml	13.67 (5.49 to 21.89)	11.30 (6.90 to 15.70)	0.577
Hemoglobin,g/L	105.63 ± 27.68	110.41 ± 30.74	0.298
Albumin,g/L	27.24 ± 5.68	28.33 ± 6.02	0.223
Leukocytes,10^9^/L	12.19 (10.41 to 13.97)	10.80 (9.47 to 12.14)	0.012
Lymphocytes,10^9^/L	1.03 (0.86 to 1.20	1.21 (0.92 to 1.50)	0.025
Neotrophils,10^9^/L	9.80 (8.29 to 11.31)	8.80 (7.64 to 9.95)	0.016
Lymphocyte/Neotrophil ratio	0.15 ± 0.05	0.20 ± 0.28	0.008
Virus infection	12(9.91%)	11(13.75%)	
Immunoglobulin			
IgG,g/L	9.95 ± 2.37	12.42 ± 3.88	0.169
IgA,g/L	1.80 ± 0.53	2.29 ± 1.04	0.156
IgM,g/L	0.90 ± 0.25	1.22 ± 0.33	0.163
IgE,ng/mL	351.97 ± 80.27	1030.29 ± 220.94	0.009
Complement component			
C3	3.27 ± 0.91	10.31 ± 2.06	0.018
C4	1.84 ± 0.46	0.25 ± 0.10	0.170
T-lymphocyte subsets			
CD3+ cells,%	61.90 ± 15.49	63.83 ± 15.29	0.647
CD4+ cells,%	33.93 ± 16.06	36.67 ± 14.93	0.521
CD4+ cell count (PCS/μl)	328.85 ± 72.23	487.86 ± 65.04	0.512
CD8+ cells,%	29.47 ± 5.42	24.38 ± 9.75	0.190
CD8+ cell count (PCS/μl)	152.75 ± 22.66	331.50 ± 95.71	0.024
CD4+/CD8+ ratio	1.79 ± 0.09	1.78 ± 0.12	0.428
B-lymphocytes			
B-lymphocyte cells,%	29.81 ± 8.89	31.33 ± 5.49	0.169
B-lymphocyte cell count (PCS/μl)	113.27 ± 29.45	601.33 ± 52.26	0.009
NK cell			
NK cells,%	9.19 ± 3.28	8.00 ± 1.67	0.708
NK cell count (PCS/μl)	56.55 ± 12.87	91.17 ± 19.79	0.134

*BMI* body mass index, *APACHE* Acute Physiology and Chronic Health Evaluation, *SOFA* Sequential Organ Failure Assessment, *CRP*,C-reactive protein, *PCT* procalcitonin

### Correlations of the lymphocyte/neutrophil ratio with disease severity and outcome

Compared with the mild group, the frequencies of lymphocyte cells were decreased in severe ARDS patients (*P* = 0.025). Moreover, the lymphocyte/neutrophil ratio decreased progressively with increasing ARDS severity (*P* = 0.001). Among non-survivors, a significantly lower lymphocyte/neutrophil ratio was found compared with that of survivors (*P* = 0.008) (Table 2), the frequencies of lymphocyte cells were lower than those in survivors (P = 0.025) (Table 2),and the frequencies of leukocytes and neutrophil cells were both higher than those in survivors (*P* = 0.012,0.016) (Table 2). There were no significant differences among the three severity groups in terms of the frequencies of leukocytes and neutrophils (Table 1).

### Alterations in inflammatory biomarkers, immunoglobulins, complement components, circulating T-lymphocyte cells, B-lymphocyte cells and NK cells in ARDS

CRP levels progressively increased with increasing ARDS severity (*P* = 0.011). The PCT level of patients with severe ARDS was higher than that of patients with mild ARDS (*P* = 0.002). Interestingly, the lymphocyte count decreased as the severity of ARDS increased (*P* = 0.025) (Table 1). In addition, compared with survivors, non-survivors were older (*P* = 0.004), and had higher leukocyte and neutrophil counts (*P* = 0.012, 0.016) and lower BMI, lymphocyte counts and lymphocyte/neutrophil ratios (*P* = 0.027, 0.025, and 0.008) (Table 2). CRP and PCT levels in the two groups were similar (Table 2).

The peripheral blood immunoglobulin IgE and complement C3 levels in patients with mild ARDS were significantly higher than those in patients with severe ARDS (*P* = 0.023, 0.019). Moreover, the levels of immunoglobulin IgE and complement C3 in non-survivors were lower than in survivors (*P* = 0.009, 0.018) (Table 2). Peripheral blood complement C4 levels in patients with mild ARDS were significantly lower than those in patients with severe ARDS (*P* = 0.026) (Table 1), but the level of complement C4 was similar between survivors and non-survivors (Table 2).

The level of peripheral blood B-lymphocyte cells was significantly lower in non-survivors than in survivors (P = 0.009), as was the level of peripheral blood CD8+ cells (*P* = 0.024), though the levels of both peripheral blood B-lymphocyte cells and CD8+ cells were similar in the three groups (Table 1). In addition, the proportions of CD3+ cells, CD4+ cells, and NK cells and the CD4+/CD8+ ratio in peripheral blood showed no significant differences among the three groups of ARDS patients stratified by oxygenation index or between the survivor and non-survivor groups.

### Correlations of lymphocytes, the lymphocyte/neutrophil ratio, immunoglobulin IgE levels, complement C3 levels, T-CD8+ lymphocyte levels and B-lymphocyte levels with disease severity and outcome

**Fig. 1:**
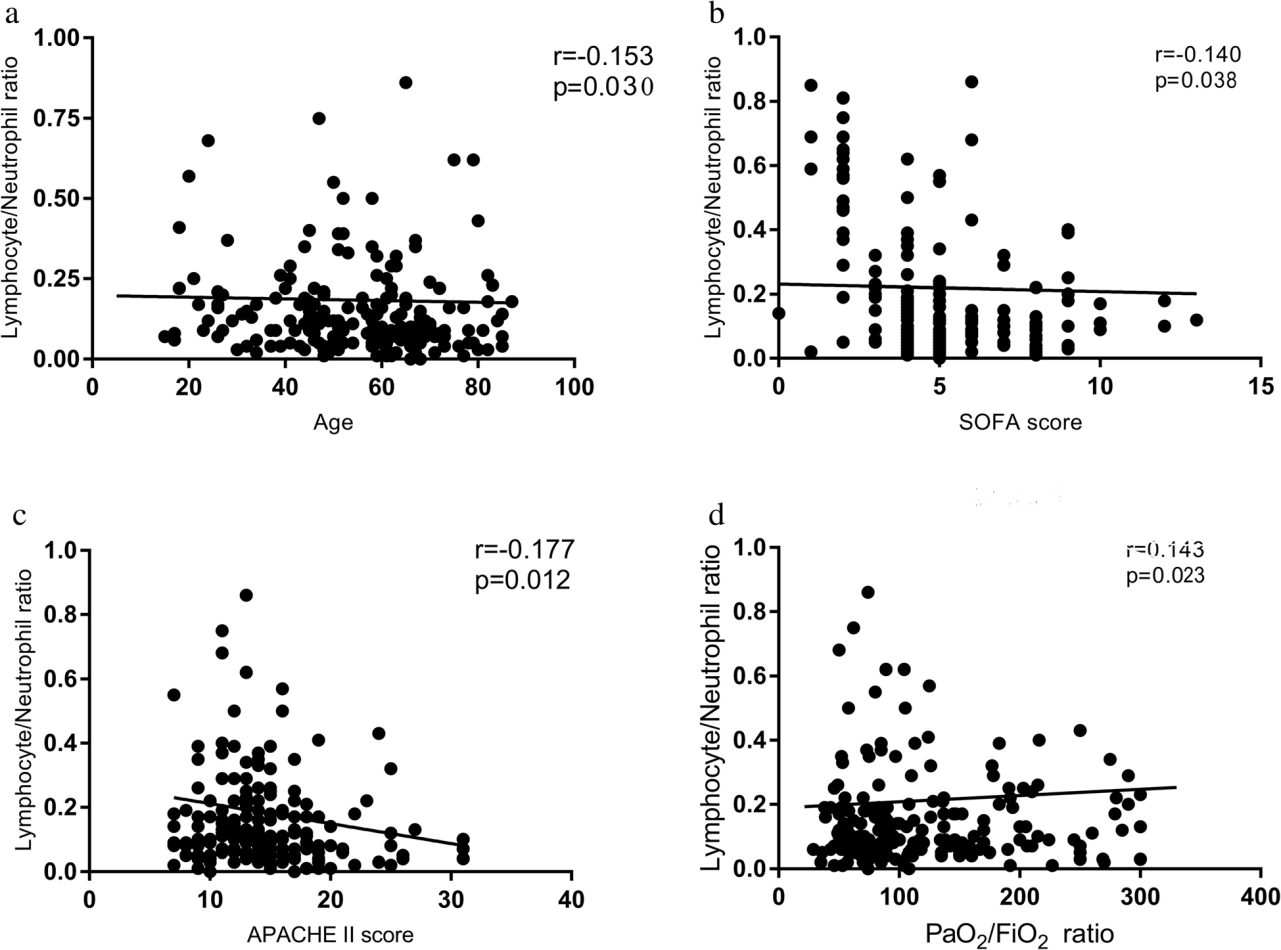
Relationships between the lymphocyte/neutrophil ratio and age, APACHE II score, SOFA score, and PaO_2_/FiO_2_ ratio in ARDS patients. Spearman rank correlation was used to assess associations between variables. The lymphocyte/neutrophil ratio correlated negatively with age (**a**), the SOFA score (**b**), and the APACHE II score (**c**) but positively with the PaO_2_/FiO_2_ ratio (D) in ARDS patients

We noted that in all ARDS patients, the lymphocyte/neutrophil ratio was moderately negatively correlated with age (r = − 0.153, *P* = 0.030, Fig.1a), SOFA score (r = − 0.140, *P* = 0.038, Fig.1b), and the APACHE II score (r = − 0.177, *P* = 0.012, Fig.1c). We also observed that, there is a moderate positive correlation between the lymphocyte/neutrophil ratio and PaO_2_/FiO_2_ ratio (r = 0.143, *P* = 0.023, Fig.1d). Moreover, significant mild positive correlations were found between the lymphocyte count and BMI (r = 0.145, *P* = 0.041), the lymphocyte count and the PaO_2_/FiO_2_ ratio (r = 0.110, *P* = 0.121), the immunoglobulin IgE level and the PaO_2_/FiO_2_ ratio (r = 0.288, *P* = 0.036), the C3 level and BMI (r = 0.342, *P* = 0.026), the T-CD8+ lymphocyte count and the lymphocyte count (r = 0.755, *P* = 0.001), the B-lymphocyte count and BMI (r = 0.588, *P* = 0.013), and the B-lymphocyte cell count and lymphocyte count (r = 0.582, *P* = 0.014).

**Fig. 2:**
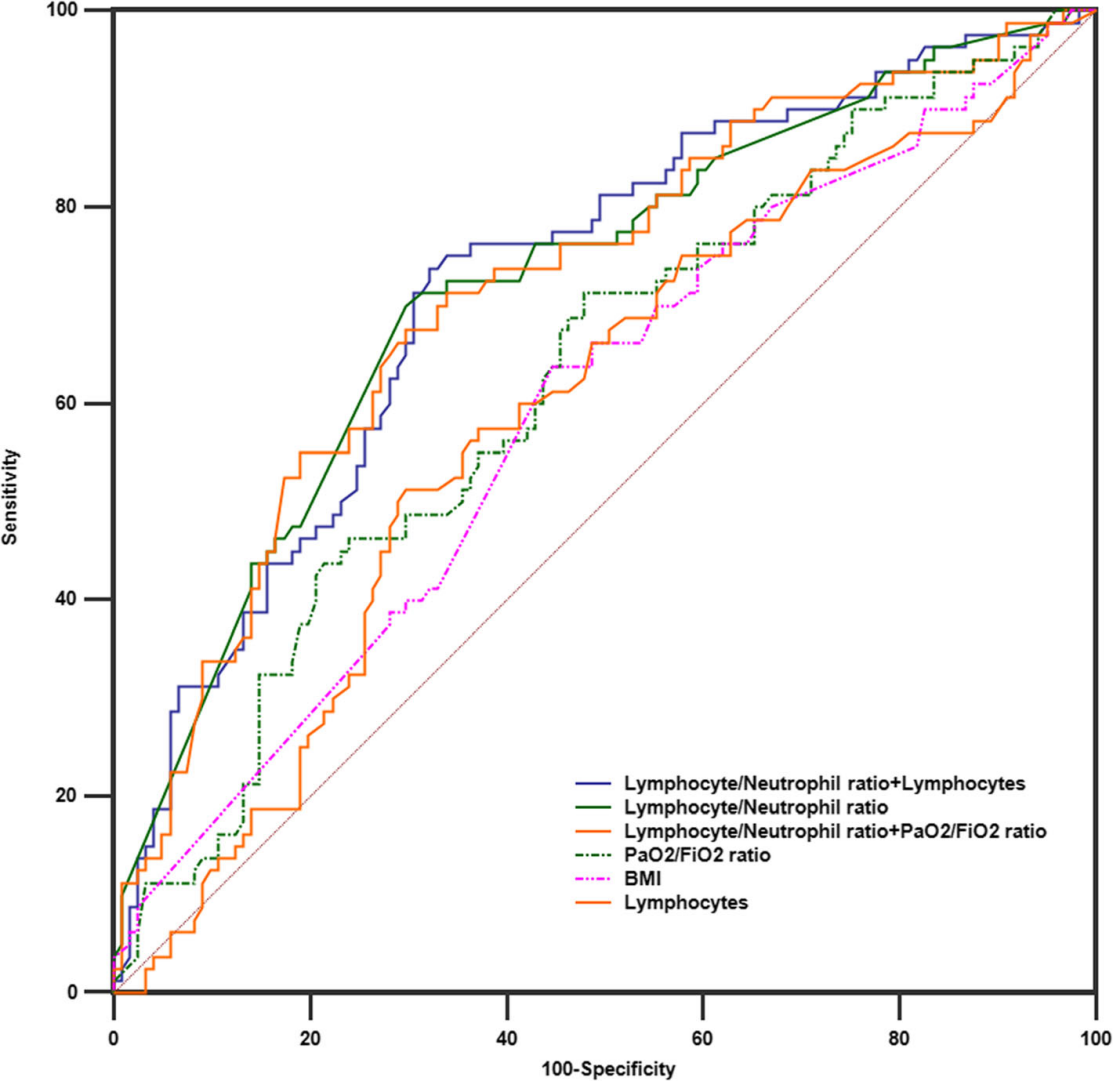
Receiver operating characteristic (ROC) curves for predicting 100-day survival in patients with acute respiratory distress syndrome (ARDS). The area under the curve (AUC) was 0.721 (95% CI 0.656 to 0.784) for the lymphocyte/neutrophil ratio, 0.625 (95% CI 0.554 to 0.692) for the PaO_2_/FiO_2_ ratio, 0.593 (95% CI 0.521 to 0.661) for the BMI, 0.592 (95% CI 0.520 to 0.660) for the lymphocyte count, 0.723 (95% CI 0.656 to 0.784) for the lymphocyte/neutrophil ratio combined with the lymphocyte count and 0.719 (95% CI 0.651 to 0.780) for the lymphocyte/neutrophil ratio in combined with the PaO_2_/FiO_2_ ratio. The AUC was 0.369 (95% CI 0.292 to 0.446) for age, 0.425 (95% CI 0.345 to 0.505) for the APACHE II score, and 0.355 (95% CI 0.278 to 0.433) for the SOFA score (not shown)

The area under the ROC curve (AUC) for the lymphocyte/neutrophil ratio for the prediction of 100-day survival in ARDS patients was 0.721 (95% CI 0.653 to 0.782) and was significantly higher than the AUC for the PaO_2_/FiO_2_ ratio alone (0.625, 95% CI 0.554 to 0.692, *P* = 0.0062,), the AUC for BMI alone (0.593, 95% CI 0.521 to 0.661, *P* = 0.0001) or the AUC for the lymphocyte count alone (0.592,95% CI 0.520 to 0.660, *P* = 0.0154) (Fig.2). The AUC for the lymphocyte/neutrophil ratio in combination with the lymphocyte count for the prediction of 100-day survival in ARDS patients was 0.723 (95% CI 0.656 to 0.784), which was larger than both the AUC for the lymphocyte/neutrophil ratio alone (*P* = 0.8601) and the lymphocyte/neutrophil ratio in combination with the PaO_2_/FiO_2_ ratio (0.719, 95% CI 0.651 to 0.780, *P* = 0.7734) (Fig.2). In predicting survival in patients with ARDS, the AUC for the lymphocyte/neutrophil ratio in combination with the lymphocyte count was significantly higher than those for the PaO_2_/FiO_2_ ratio alone (*P* = 0.0060), BMI alone (*P* = 0.0001), and lymphocyte count alone (*P* = 0.0067), and the AUC for the lymphocyte/neutrophil ratio in combination with the PaO_2_/FiO_2_ ratio was significantly higher than those for the PaO_2_/FiO_2_ ratio alone (*P* = 0.0014), BMI alone (P = 0.0001), and lymphocytes alone (*P* = 0.0162).

A cut-off value of the lymphocyte/neutrocyte ratio of > 0.0537 was used to predict the survival of ARDS patients. The sensitivity was 83.8%, and the specificity was 80.2%. The positive likelihood ratio was 4.23, and the negative likelihood ratio was 0.20. Moreover, a leukocyte count cut-off of > 0.415 (10^9^/L) was used to predict the survival of patients with ARDS. The sensitivity was 87.5%, and the specificity was 81.0%. The positive likelihood ratio was 4.61, and the negative likelihood ratio was 0.15.

### Predictors of 28-day and 100-day mortality in patients with ARDS

**Fig. 3:**
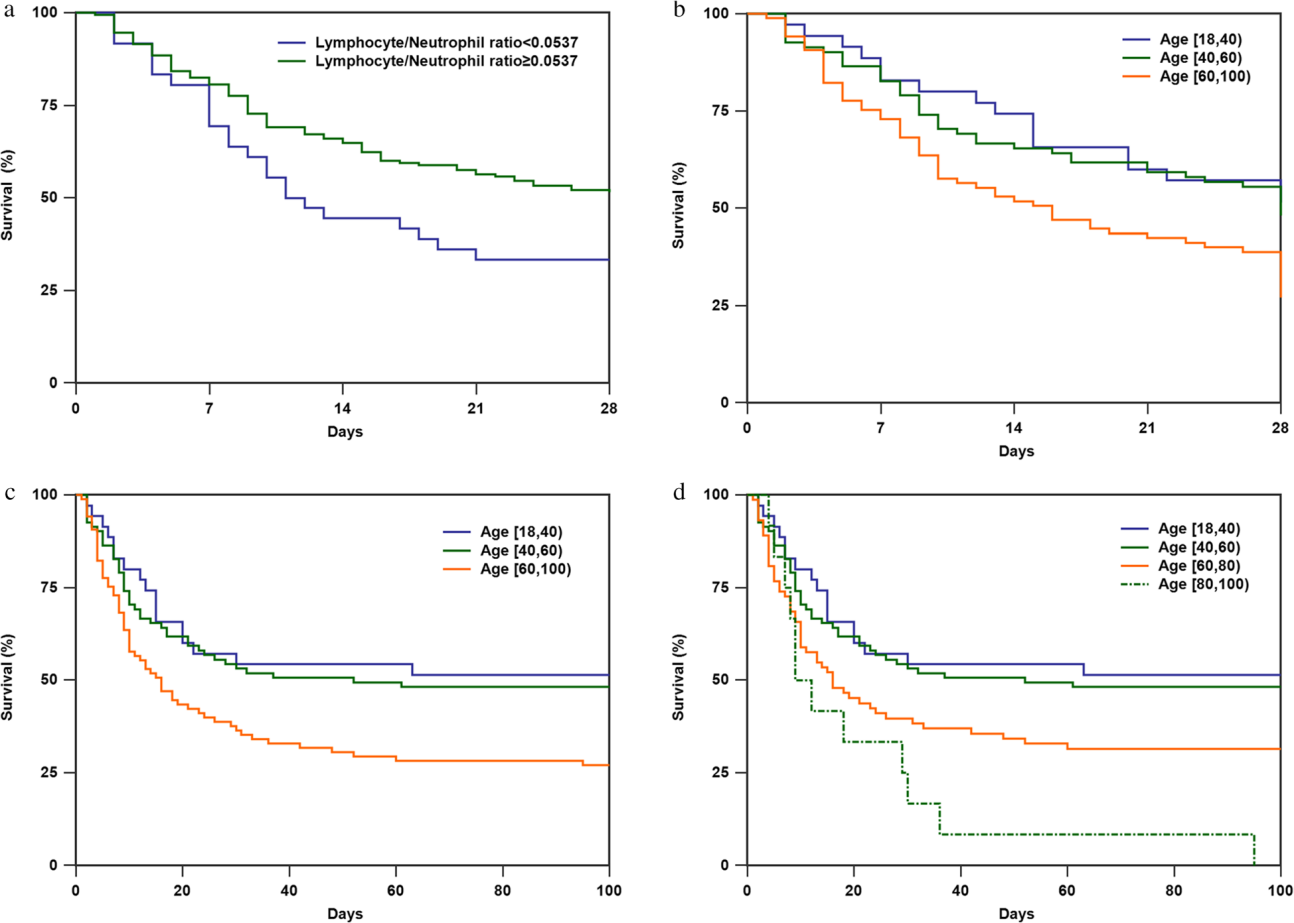
Kaplan-Meier survival curve for patients with ARDS using the cut-off values for the lymphocyte/neutrophil ratio and age obtained by ROC analysis. Log-rank test (*P* = 0.0283) (**a**), (*P* = 0.0064) (**b**), (*P* = 0.0057) (**c**), and (*P* = 0.0029) (**d**)

Table 3 shows that age (per log_10_ years) (OR = 1.269, *P* = 0.019), BMI < 24 (OR = 1.665, *P* = 0.015), SOFA score (OR = 1.287, *P* = 0.002), leukocyte count< 0.415 (10^9^/L) (OR = 1.671, *P* = 0.042), and lymphocyte/neutrophil ratio (OR = 2.132, *P* = 0.009) were independent predictors of 100-day mortality in ARDS patients. Moreover, ARDS patients with a lymphocyte/neutrophil ratio < 0.0537 had a higher 28-day mortality rate than did those with a lymphocyte/neutrophil ratio > 0.0537 (*P* = 0.0283, Fig. 3a). Furthermore, 28-day and 100-day mortality rates were significantly lower in those under 40 years old and 40–60 years old than in those over 60 years old age (*P* = 0.0064, 0.0057, Fig. 3b, c). The 100-day mortality rate was significantly higher in those over 80 years old than in those under 40 years old, 40–60 years old and 60–80 years old (*P* = 0.0029, Fig. 3d).

**Table 3:** Logistic regression analysis of the prediction of mortality for patients with acute respiratory distress syndrome (ARDS)

Variables	Univariate analysis		Multivariate analysis	
	Odds ratio (95% CI)	*P*-value	Odds ratio (95% CI)	*P*-value
Age,per log_10_(years)	1.269(1.040,1.548)	0.019	2.982(2.073,4.654)	0.007
BMI,< 24	1.665(0.883,3.137)	0.015		
APACHE II score,per point	1.016(0.940,1.098)	0.059		
SOFA score,per point	1.287(1.098,1.509)	0.002	2.560(1.457,5.430)	0.005
PaO_2_/FiO_2_,per log_10_(mmHg)	0.652(0.280,1.004)	0.067		
PCT,per log_10_(ng/mL)	1.028(0.797,1.810)	0.063		
CRP,> 150(mg/L)	1.256(0.618,2.553)	0.059		
Lymphocytes,< 0.415 × 10^9^/L	1.671(1.252,1.787)	0.042		
Lymphocyte/Neutrophil ratio,< 0.0537	4.137(1.452,6.832)	0.002	3.726(2.754,5.195)	0.003

*BMI* body mass index, *APACHE* Acute Physiology and Chronic Health Evaluation, *SOFA* Sequential Organ Failure Assessment, *PCT* procalcitonin, *CRP* C-reactive protein

## Discussion

In this study, we found associations between age, BMI, the SOFA score, and the lymphocyte/neutrophil ratio at ICU admission and clinical outcomes in patients with ARDS. Age (per log_10_ years), BMI < 24, the SOFA score (per point) and the lymphocyte/neutrophil ratio were independent risk factors for predicting 100-day mortality in ARDS patients. Another discovery was that the lymphocyte/neutrophil ratio and age were related to ICU mortality and hospital mortality. We also found associations between the baseline lymphocyte/neutrophil ratio and age, the SOFA score, the APACHE II score, the PaO_2_/FiO_2_ ratio, and the severity of ARDS according to the Berlin classification. The lymphocyte/neutrophil ratio may help predict prognosis for ARDS patients with a high immunologic risk. Our study is a longitudinal clinical outcome study of ARDS patients, and the results demonstrate the predictive significance of the lymphocyte/neutrophil ratio.

During the past decade, there have been a few investigations addressing the potential function of the lymphocyte/neutrophil ratio, which remains a useful test for the diagnosis of tuberculous pleuritis [19] and acts as an early biomarker for predicting acute rejection after heart transplantation [20]. Previous studies have focused on the poor prognosis of patients with severe lymphopenia from the first day of ICU admission [21]. In our study, peripheral blood lymphopenia was very common in ARDS patients without typical underlying diseases, causing immunosuppression. The number of peripheral blood lymphocytes decreased significantly in patients with severe ARDS, and in non-survivors also. Moreover, the lymphocyte/neutrophil ratio progressively decreased with increasing ARDS severity, and a significantly lower lymphocyte/neutrophil ratio was found in non-survivors than in survivors.

A decrease in the lymphocyte/neutrophil ratio is due to a decrease in the lymphocyte count and an increase in the neutrophil count. In our study, ARDS patients with a lymphocyte/neutrophil ratio < 0.0537 had a higher 28-day mortality rate than did those with a lymphocyte/neutrophil ratio ≥ 0.0537. The lymphocyte/neutrophil ratio may reveal the balance between lymphocyte and neutrophil counts. Lymphocytes are important immune cells involved in response to ARDS and in prognosis. Multivariate analysis showed that a decrease in the lymphocyte count was associated with a 2.32–3.76-fold increase in the risk of death among patients with or without septic shock [22]. In addition, both B-lymphocyte and CD8 + T-lymphocyte counts correlated positively with peripheral blood lymphocyte counts in our study, these counts in non-survivors were significantly lower than those in survivors, and the findings suggest increased risk of death in ARDS patients when the lymphocyte count decreases below a certain value. Neutrophils are another type of immune cell involved in the process of sepsis, and a relative increase in the total number of circulating neutrophils and the percent increase in neutrophils with immature morphology are also closely related to sepsis [23, 24]. Compared with counts in survivors, higher neutrophil counts were found in patients who eventually died as a result of sepsis-induced ARDS, and excessive accumulation of neutrophils in patients with ARDS may therefore contribute to disease progression [6, 8]. Therefore, the combination of lymphopenia and neutrophilia contributes to the outcome of ARDS, which may explain why the lymphocyte/neutrophil ratio in our study was a strong independent predictor of prognosis.

Although the clinical scores, such as APACHE II [25], SOFA [26] and the PaO_2_/FiO_2_ ratio [27], have been widely used to predict the results of clinical practice patients of ARDS. We further found that the lymphocyte/neutrophil ratio AUC was higher than the PaO_2_/FiO_2_ ratio, BMI, APACHE II score, and SOFA score alone in predicting 100-day survival in ARDS patients. In combination with the lymphocyte count slightly increased the AUC of the lymphocyte/neutrophil ratio in predicting 100-day survival, yet there was no difference compared with the lymphocyte/neutrophil ratio alone. Moreover, ARDS patients with a lymphocyte/neutrophil ratio ≥ 0.0537 had a lower 28-day mortality rate, and significantly moderate negative correlations were found between the lymphocyte/neutrophil ratio and age, the SOFA score, and the APACHE II score. In addition, a moderate positive correlation between the lymphocyte/neutrophil ratio and the PaO_2_/FiO_2_ ratio was been noted. These results suggest that the lymphocyte/neutrophil ratio can comprehensively and plausibly reflect the patient’s physiological, pathophysiological and respiratory oxygenation index status. Taken together, our findings strongly suggest that the lymphocyte/neutrophil ratio is a potential indicator and good indicator for prognosis evaluation among ARDS patients.

There have been many clinical studies on ARDS patients with typical immunodeficiency [13, 28], and immunodeficiency in ARDS patients is usually atypical. Although it does not reach the level found in typical immunodeficiency, there is already a degree of immune impairment. Nonetheless, there are few studies on atypical immune deficiency or impaired immunity in ARDS patients, and there is no uniform scale or biomarker to measure immune impairment in ARDS patients and its relationship with prognosis. A consensus has not been reached regarding whether viral infection causes immune deficiency, though it is commonly thought to induce immune impairment but not to the extent of immune deficiency. In our study, there was no significant difference in viral infection status between the survivor and non-survivor groups. However, we did observe that the lymphocyte/neutrophil ratio in the survivor group were higher than those in the non-survivor group. We hypothesize that a low lymphocyte/neutrophil ratio may be a marker of this atypical immunodeficiency in ARDS patients, affecting their prognosis. There are no recognized biomarkers to date that can be used to identify the immune status of ARDS patients. Accordingly, we propose a new biomarker for the identification of atypical immune status in patients with ARDS. This status may be due to abnormal inhibitors that have been be activated. We hope that by observing the association of the early lymphocyte/neutrophil ratio with prognosis may result in better detection and more timely treatment of an abnormal immune status.

Our findings must be understood in view of the following limitations. First, this study had a relatively small sample size, even though it was the first study to explore the prognostic value of the lymphocyte/neutrophil ratio for prognosis in ARDS patients. Second, there may be a selection bias because only patients for whom absolute lymphocyte and neutrophil counts were measured soon after ARDS diagnosis were included. Third, the current study was not pre-specified but is a post hoc analysis from a retrospective controlled trial. Therefore, further prospective studies are needed.

## Conclusions

We found age (per log_10_ years), BMI < 24, the SOFA score, the lymphocyte count, and the lymphocyte/neutrophil ratio to be independent predictors of 100-day mortality in patients with ARDS. We also observed a moderate negative correlations between the lymphocyte/neutrophil ratio and age, the SOFA score, and the APACHE II score, and a significant mild positive correlation between the lymphocyte count and BMI was also found. However, we detected a moderate positive correlation between the lymphocyte/neutrophil ratio and the PaO_2_/FiO_2_ ratio in all patients with ARDS. The AUC was greatest for the lymphocyte/neutrophil ratio combined with the lymphocyte count for the prediction of 100-day survival in ARDS. In addition, the 28-day mortality rate of ARDS patients with a lymphocyte/neutrophil ratio < 0.0537 was higher than that with a ratio of lymphocyte/neutrophil ≥0.0537. The lymphocyte/neutrophil ratio was also revealed to be a strong and independent predictor of prognosis in ARDS patients, particularly in those with atypical immunodeficiency.

## Data Availability

The datasets used and analysed during the current study are available from the corresponding author on reasonable request.

## Abbreviations

ARDS: Acute respiratory distress syndrome
APACHE: Acute Physiology and Chronic Health Evaluation
AUC: Area under the curve
BALF: Bronchoalveolar lavage fluid
BMI: Body mass index
CI: Confidence interval
CRP: C-reactive protein
ICU: Intensive care unit
LDH: Lactate dehydrogenase
MOF: Multiple organ failure
OR: Odds ratio
PCT: Procalcitonin
ROC: Receiver operating characteristic
SAPS: Simplified Acute Physiology Score
SD: Standard deviation
SOFA: Sequential Organ Failure Assessment
